# Emotion-focused Cognitive-Behavioral Therapy for externalizing disorders in children and adolescents : an attempt to resolve emotion regulation difficulties

**DOI:** 10.1192/j.eurpsy.2022.1124

**Published:** 2022-09-01

**Authors:** N. Arfaoui, M. Hajri, Z. Abbes, S. Halayem, A. Bouden

**Affiliations:** 1 Razi Hospital, Child And Adolescent Psychiatry, Manouba, Tunisia; 2 Razi Hospital, Child And Adolescent Psychiatry, manouba, Tunisia; 3 Razi hospital, Child And Adolescent Psychiatry, manouba, Tunisia

**Keywords:** externalizing behaviors, Children, emotion regulation

## Abstract

**Introduction:**

Deficient emotion regulation is a common and impairing area of difficulty among children and adolescents with externalizing disorders. Emotion focused cognitive behavioral therapy ECBT is a form of CBT that is suggested to be employed to improve dysregulation of anxiety and other kind of emotions in anxious youth.

**Objectives:**

Examine the efficacy of an Emotion-focused Cognitive-Behavioral Therapy (ECBT) inspired program on emotional regulation difficulties and behavioral problems in children and adolescents with externalizing disorders

**Methods:**

We conducted a cross-sectional comparative experimental study. Subjects were 50 patients exhibiting behavioral disorders aged 9 to 18 years , with a diagnosis of attention-deficit/hyperactivity disorder (ADHD), oppositional defiant disorder (ODD), and conduct disorder (CD).Participants were assigned to ECBT and control groups. ECBT group contributed in 12-h weekly sessions within ECBT-inspired program. The control group received standard care. To assess emotion-related competencies, children were administered the emotion regulation questionnaire (ERQ-CA) and the alexithymia questionnaire for children(AQC). Parents completed the Child Behavior Checklist(CBCL) to measure youth externalizing problems. Tests were administered in pre- and post-test to all subjects. Both groups were matched for age, sex and educationnal level. Comparison of pre- and post-test results was performed using the Student’s t-test.

**Results:**

ECBT demonstrated a significant difference in the reduction of behavioral problems. ECBT effectively increased adaptive emotion regulation strategies (cognitive reappraisal) in the post-test. ECBT also reduced alexithymia scores, particularly difficulty identifying feelings, and externally oriented thinking.

**Conclusions:**

ECBT demonstrated promising initial effectiveness in addressing emotion regulation deficits of children with externalizing behaviors

**Disclosure:**

No significant relationships.

